# Optimized processing and formulation for retaining the sensory and nutritional quality of mustard leaf tea

**DOI:** 10.1016/j.fochx.2025.102515

**Published:** 2025-05-01

**Authors:** Xuena Yu, Hongmei Di, Yatian Zhao, Ruobin Liu, Yiqing Wang, Yuxin Lu, Kehao Liang, Zhifeng Chen, Jie Ma, Zhongrong Guan, Zhi Huang, Yi Tang, Jingyi Xu, Huanxiu Li, Qian Tang, Yiting Chen, Jinlin Bian, Fen Zhang, Bo Sun

**Affiliations:** a College of Horticulture, Sichuan Agricultural University, Chengdu 611130, China; b College of Biology and Agriculture Technology, Zunyi Normal University, Zunyi 563000, China; c Bijie Institute of Agricultural Science, Bijie 551700, China; d Southeast Chongqing Academy of Agricultural Sciences, Chongqing 408000, China; e Department of Plant and Environmental Sciences, Faculty of Science, University of Copenhagen, Højbakkegård Allé 13, DK-2630 Taastrup, Denmark

**Keywords:** *Brassica juncea*, Herbal tea, Processing technology, Blended tea, Sensory evaluation, Health-promoting compounds

## Abstract

The potential of mustard (*Brassica juncea*) leaves as a raw material for herbal tea remains unexplored. This study aimed to evaluate the suitability of mustard leaves for tea production and optimize processing and formulation. Four mustard varieties were evaluated using traditional green tea processing techniques, demonstrating that purple-leaf mustard is an excellent raw material for herbal tea, both in terms of sensory properties and bioactive compounds. Next, eight processing methods for purple-leaf mustard were employed. The mustard leaf tea, processed using a combination of steaming fixation, rolling, and vacuum freeze-drying technology, demonstrated effective preservation of health-promoting compounds while maintaining a desirable flavor profile. Blending mustard leaf tea with Maofeng green tea or Tieguanyin oolong tea further enhanced its aroma and taste. This study developed a novel herbal tea using purple-leaf mustard and provides a reference for producing high-quality herbal teas with a unique flavor profile and health-promoting compounds.

## Highlights

• Developed a novel herbal tea with unique sensory properties and health benefits.
• The optimization of processing techniques to preserve bioactive compounds.
• Blended mustard leaf tea with traditional tea enhanced the flavors.

## 1 Introduction

Tea is one of the top three non-alcoholic beverages consumed worldwide, particularly in China, where it has become a daily habit for people of all ages (Pan et al., 2022). However, traditional tea is made from the leaves and buds of *Camellia sinensis* and contains 3 % to 5 % caffeine, which can be problematic for some individuals (Samanta, 2022). Caffeine stimulates the cerebral cortex, inducing central nervous system excitation, and can irritate the gastrointestinal tract, leading to sleeplessness in certain individuals (Ye et al., 2007). Herbal teas have become a choice for many tea lovers, as a healthy alternative with low calorie and decaffeinated (Gramza-Michałowska, 2014).

Herbal tea is brewed from the leaves, flowers, seeds, fruits, stems, or roots of various plant species, rather than from *Camellia sinensis*, and has been used for health care and disease prevention for thousands of years in many cultures (Aoshima, Hirata, & Ayabe, 2007; Deetae, Parichanon, Trakunleewatthana, Chanseetis, & Lertsiri, 2012; Wei et al., 2024). In recent decades, the global consumption of herbal drinks has increased, with more plants with medicinal properties being used in herbal teas. According to available statistics, over 759 species from 86 families and 209 genera are used to make herbal teas in China (Fu et al., 2018). Common herbal teas are made from ginger, chamomile, cinnamon, rosehip, peppermint, yerba mate, sage, rooibos, or combinations of herbs (Ruchika & Sehgal, 2022). Since herbal teas are prepared from a wide variety of plant materials, they offer unique flavors and rich tastes, giving consumers a broad range of choices. Herbal teas are becoming increasingly popular in many countries, and in Germany, their consumption (including fruit tea) even surpasses that of traditional tea (Sousa, Pádua, Gonçalves, Ribeiro, & Leal, 2024).

The flavor and quality of herbal tea largely depend on both the raw materials and the processing techniques used. In the past, the majority of herbal teas were coarse dried (by methods such as sun-dried, dried in the shade, roasted, and baked) before use (Fu et al., 2018). However, as consumer demand for the sensory and nutritional qualities of herbal teas has increased, processing techniques have been continuously upgraded for different types of herbal tea (Liu, Ahmed, & Long, 2013). For example, *Bletilla striata* tea processed by freeze-drying retained its original aroma and flavor and received the highest sensory evaluation scores (Han et al., 2023). The processing of DuZhong tea involves enzyme deactivation, rolling, drying, final panning, and sometimes fermentation to reduce bitterness and astringency (Shi et al., 2019; Shi, Luo, Zhong, Hu, & Zhang, 2022). Additionally, the flavonoid and phenolic content of mulberry leaf tea increased when using green and black tea techniques (Panyatip et al., 2022). Therefore, developing appropriate processing methods is essential to improving the flavor and quality of herbal teas and expanding their market share.

However, with the rapid expansion of the herbal tea market, attention must be given to the quality, efficacy, and safety of these products. For example, there may be safety risks associated with using medicinal plants in herbal teas (Halegoua-DeMarzio & Navarro, 2024; Lal, Chanotiya, & Kumar, 2023). Some herbal teas, such as *Lithospermum officinale*, have been reported to contain pyrrolizidine alkaloids, which may cause hepatotoxicity with long-term consumption (Wiedenfeld, 2011). Additionally, some producers and distributors have begun adding sweeteners to mask the bitterness of herbal drinks, which increases calorie content and alters their health benefits (Liu et al., 2013). Furthermore, the plant resources used to produce herbal teas are becoming threatened in certain regions. Many herbal tea plants are wild-harvested, and overharvesting could occur as commercialization continues (Fu et al., 2018; Liu et al., 2013). Therefore, the development of new herbal tea products using non-toxic species with health-promoting compounds, pleasant flavor, and the ability to be widely cultivated, such as vegetables rather than medicinal plants, would support safer production and ensure the traceability of raw materials.

Leaf mustard (*Brassica juncea*) is one of the most commonly consumed vegetables in the Brassica family (Li et al., 2023). It contains a variety of nutrients and bioactive compounds, such as ascorbic acid, carotenoids, phenolics, and glucosinolates (GSLs) (Di et al., 2023; Zhang et al., 2021). Especially GSLs, mainly distributed in the order Brassicales, not only contribute to plant defense and the characteristic flavor of mustard but also produce hydrolysis products that have been recognized for their role in reducing the risk of various cancers (Sundaram et al., 2022). Additionally, leaf mustard possesses diverse germplasm resources, including different leaf color variations. Purple leaf mustard, for instance, is rich in anthocyanins, which are natural food colorants and play a crucial role in antioxidant activities (Castañeda-Ovando, de Lourdes Pacheco-Hernández, Páez-Hernández, Rodríguez, & Galán-Vidal, 2009). Despite being a highly nutritious and health-promoting vegetable, the development and utilization of leaf mustard products remain limited. Currently, leaf mustard is primarily consumed fresh or processed into fermented foods such as *suancai* and *paocai* (Di et al., 2023; Zhang et al., 2021). However, fermentation significantly reduces the phytochemical content in mustard (Di et al., 2023; Liu, Zhang, Zhang, Xin, & Wu, 2022). Therefore, developing mustard products that retain more of their original metabolites, such as herbal tea, would enhance the value of mustard products and drive innovation in the mustard industry to meet growing market demand.In this study, four varieties of leaf mustard were processed into herbal tea using green tea production techniques. The most suitable mustard variety for tea production was identified through sensory evaluation and component analysis. The process technology of mustard leaf tea was then explored, resulting in the development of a new, unique, and nutritious herbal tea. Additionally, the mustard leaf tea was blended with traditional teas to create a product with a pleasant aroma and coordinated taste. This study successfully developed a novel herbal tea using purple leaf mustard, providing a reference for the creation of high-quality herbal teas with distinctive flavors and health-promoting compounds.

## 2 Materials and methods

### 2.1 Varieties and processing technology selection of mustard leaf tea

#### 2.1.1 Materials

Four varieties of leaf mustard (*Brassica juncea* var. *rugosa*)—L6 (green leaf), Y3 (yellow-green leaf), L1 (green leaf with a purple reticular pattern), and L2 (purple leaf)—were cultivated in Yibin City, Sichuan Province, China. Samples were collected in January 2024. Thirty fully developed plants of each variety were harvested and transported to the teaching practice tea factory of Sichuan Agricultural University.

#### 2.1.2 Mustard tea raw material screening

Green tea processing techniques were employed to produce mustard leaf tea. The mature leaves of L6, Y3, L1 and L2 were washed and air-dried. The leaves were then cut into 2 cm squares and fixed using a microwave oven (2450 MHz, 700 W) for 15 s, repeated five times, to rapidly inactivate endogenous enzymes. The leaves were hand-rolled into ball shapes and dried with hot air for 30 min, followed by 20 min of cooling, with this cycle repeated six times*.* The dried mustard leaf teas were stored at −20 °C for subsequent analysis of health-promoting compounds and sensory quality evaluation.

#### 2.1.3 Mustard tea processing technology screening

Using purple leaf mustard (L2) as the raw material, several key steps in the mustard leaf tea processing technique were compared, including fixation, rolling, and drying methods. For fixation, microwaving and steaming were evaluated. The microwaving method followed the procedure described previously, while the steaming method involved treating the leaves with 100 °C steam for 1 min. For rolling, hand-rolling into ball shapes was compared with non-pressure rolling. The drying methods compared were vacuum-freeze drying and hot-air drying. In vacuum-freeze drying, the samples were pre-frozen at −80 °C and then dried in a vacuum freeze dryer, while the hot-air drying method followed the previously described procedure. An orthogonal test was designed, resulting in eight mustard leaf tea products with different processing combinations (Fig. 2A):

(1) MNV was leaves of L2 processed by Microwaving fixation, next Non-pressure rolling and Vacuum-freeze drying;

(2) MNH was leaves of L2 processed by Microwaving fixation, next Non-pressure rolling and Hot-air drying;

(3) MBV was leaves of L2 processed by Microwaving fixation, next Ball shaping by hand-rolling and Vacuum-freeze drying;

(4) MBH was leaves of L2 processed by Microwaving fixation, next Ball shaping by hand-rolling and Hot-air drying;

(5) SNV was leaves of L2 processed by Steaming fixation, next Non-pressure rolling and Vacuum-freeze drying;

(6) SNH was leaves of L2 processed by Steaming fixation, next Non-pressure rolling and Hot-air drying;

(7) SBV was leaves of L2 processed by Steaming fixation, next Ball shaping by hand-rolling and Vacuum-freeze drying;

(8) SBH was leaves of L2 processed by Steaming fixation, next Ball shaping by hand-rolling and Hot-air drying.

Samples from fresh, fixated, rolled, and dried leaves were collected in triplicate for evaluation. The samples were lyophilized and stored at −20 °C for subsequent nutritional component analysis.

#### 2.1.4 Mustard tea blended with traditional Chinese teas

The mustard leaf tea processed by SBV was blended in a 1:1 ratio with 14 traditional Chinese teas, including four classic green teas (Yuzhen, Maofeng, Taiping Houkui, and Mengding Ganlu), white tea (Bai Mu Dan), yellow tea (Mengding Huangya), oolong tea (Tieguanyin), three black teas (Keemun, Yingde, and Chuanhong), dark tea (Yaan Tibetan tea), and three scented teas (Osmanthus black tea, Rose black tea, and Jasmine green tea). For evaluating sensory quality, the blended tea was brewed in water at 100 °C for 5 min, with a tea-to-water ratio of 1:50.

### 2.2 Evaluation of sensory quality

Each mustard leaf tea sample (1:50, *w*/*v*) was brewed with boiling water for 5 min, and 2 mL of the infusion was poured into a teacup. Ten scholars specializing in tea science, who had received training in sensory evaluation, assessed the sensory qualities of the mustard leaf tea according to the national standard method for sensory review of teas (GB/T 23776–2018) (Gong et al., 2018). Informed consent was obtained from all participating reviewers to the sensory evaluation. The assessors conducted an objective and comprehensive evaluation of the sensory attributes. As for mustard leaf tea, the sensory evaluation followed a weighted scoring system based on four key attributes: appearance (10 %), liquor color (10 %), aroma (40 %), and taste (40 %), yielding maximum sub-scores of 10, 10, 40, and 40 points respectively (total possible score: 100 points). For the blended teas, the distinct morphological characteristics of traditional tea make appearance evaluation difficult to standardize. Therefore, a comprehensive evaluation was independently scored on liquor color (20 %), aroma (40 %), and taste (40 %), yielding maximum sub-scores of 20, 40, and 40 points, respectively (total possible score: 100 points). This weighting scheme was established a priori by tea evaluation experts to reflect the relative importance of each attribute in the overall tea quality assessment. The sensory evaluation method and scoring coefficient were consensus from the panel.

### 2.3 Anthocyanin content

Anthocyanin content was determined according to previous reports (Zhang et al., 2014). Fifty milligrams of sample powder was vortexed with 3 mL of methanol/water/acetic acid (85:15:0.5; *v*/*v*/*v*), followed by ultrasonication for 20 min at 25 °C sonicated, and leached overnight at 4 °C in the absence of light. The mixture was subsequently centrifuged at 10,000 ×*g* for 10 min, and the supernatant was collected for anthocyanins analysis using an Agilent 1260 high-performance liquid chromatography (HPLC) instrument equipped with a VWD detector. The mobile phase consisted of (A) 5 % (*v*/v) formic acid aqueous solution and (B) acetonitrile (HPLC grade), with a constant flow rate of 0.8 mL min^−1^. Detection was carried out at 530 nm, and the gradient elution program was optimized as follows: 0 min, 90 % A; 15 min, 87 % A; 45 min, 80 % A; 50 min, 77 % A. Anthocyanins were quantified according to Cyanidin-3,5-O-diglucoside chloride (HPLC purity ≥95 %, Yuanye Bio-Technology Co., Ltd., Shanghai, China).

### 2.4 Chlorophyll and carotenoid content

Fifty milligrams of tea powder was homogenized and extracted with 25 mL of acetone. The samples were sonicated for 20 min and centrifuged at 4000 *g* at room temperature for 5 min. The supernatant was filtered through 0.22 μm nylon syringe filters, and the analysis of chlorophylls and carotenoids was carried out using an Agilent 1260 instrument with a variable wavelength detector (VWD) (Agilent Technologies, Inc., Palo Alto, USA). Samples (10 μL) were separated at 30 °C on a Waters C18 column (150 × 3.9 mm i.d.; 4 μm particle size) using (A) isopropanol and (B) 80 % acetonitrile–water at a flow rate of 0.5 mL min^−1^. The linear change is as follows: 0 min, 0 % A; 45 min, 100 % A. Absorbance was detected at 448 and 428 nm. Chlorophylls (a and b) (HPLC≥95 %) and carotenoids [neoxanthin (HPLC≥90 %), violaxanthin (HPLC≥90 %), lutein (HPLC≥90 %), and β-carotene (HPLC≥98 %)] were quantified according to the respective standard calibration curves, and the standards were obtained from Solarbio Science & Technology Co., Ltd. (Beijing, China) (Sun et al., 2021).

### 2.5 GSL composition and content

The GSL composition and content were determined following established protocols (Di et al., 2023). Freeze-dried samples (100 mg) were boiled in 5 mL water for 10 min. The supernatant was collected after centrifugation, and the residues were washed once with water, centrifuged at 7000 *g* for 5 min, and then combined with the previous extract. The aqueous extract (1 mL) was applied to a DEAE-Sephadex A-25 column (Sigma Chemical Co., Saint Louis, USA). The GSLs were converted into their desulpho analogues by overnight treatment with 100 μL of 0.1 % aryl sulphatase (Sigma Chemical Co., Saint Louis, USA), and the desulphoglucosinolates were eluted with 1 mL water. HPLC desulphoglucosinolate analysis was performed using an Agilent 1260 HPLC instrument equipped with a VWD detector (Agilent Technologies, Inc., Palo Alto, USA). Samples were separated at 30 °C on a Waters Spherisorb C18 column (250 × 4.6 mm i.d.; 5 μm particle size) using (A) acetonitrile and (B) water at a flow rate of 1.0 mL min^−1^. The linear change is as follows: 0–5 min, 1.5 % A; 20–30 min, 20 % A. Absorbance was detected at 226 nm. Analyte content was calculated using ortho Nitrophenyl β-D-galactopyranoside (oNPG) (≥98 %) (Sigma Chemical Co., Saint Louis, USA) as the internal standard (Sun et al., 2018).

### 2.6 GSL breakdown products composition and content

The composition and content of GSL breakdown products (GBPs) were determined using gas chromatography–mass spectrometry (GC–MS) based on established methods (Di et al., 2023). Freeze-dried samples (100 mg) were ground in 2 mL of double-distilled water (ddH_2_O) in the presence of 0.2 μmol of the internal standard benzonitrile (Sigma-Aldrich Chemie GmbH, Germany). The extracts were incubated at 25 °C for 1 h. Following this, 5 mL of dichloromethane (LABOR, China) was added and vortexed, then allowed to sit for 20 min at 25 °C. The mixture was centrifuged, and the lower dichloromethane layer was collected and transferred into a vial. The analysis was conducted using an Agilent 7890B—5977B gas chromatograph-mass spectrometer (Agilent Technologies, Waldbronn, Germany) equipped with an HP-5 ms column (30 m × 0.25 mm × 0.25 μm film) to identify the GBP. The injection was performed in splitless mode at 250 °C, with a carrier gas (hydrogen, 99.99 %) flow rate of 1 mL min^−1^. The column temperature was initially set at 35 °C for 3 min, then increased to 250 °C at a ramp rate of 10 °C/min, maintained for 10 min. All compounds were scanned in the mass-to-charge ratio range of 30–350 *m*/*z*. Peaks were identified by comparing their mass spectra with literature data (Wang et al., 2019). Quantification was based on the peak area relative to that of the internal standard, benzonitrile (Zeng et al., 2021).

### 2.7 Glucose, fructose, and sucrose content

The contents of glucose, fructose, and sucrose were determined and analyzed as previously described (Sun et al., 2021). Freeze-dried samples (100 mg) were added to 5 mL of ddH_2_O and homogenized for 1 min. The mixture was then extracted in a water bath at 80 °C for 30 min. The supernatant was collected after centrifugation at 8000 *g* at room temperature for 5 min, and filtered through a 0.45 μm cellulose acetate filter, and then analyzed by HPLC using an Agilent 1260 instrument equipped with a refractive index detector (Agilent Technologies, Inc., Palo Alto, USA). Samples (10 μL) were separated at 35 °C on an Agilent ZORBAX carbohydrate column (250 × 4.6 mm i.d.; 5 μm particle size) using 80 % acetonitrile at a flow rate of 1.0 mL min^−1^. Contents of individual soluble sugars [glucose (≥99.5 %), fructose (≥99.0 %), and sucrose (≥99.0 %)] were determined using the standard calibration curves for each sugar (Sangon Biotech Co., Ltd., shanghai, China), and the content of total soluble sugars was calculated by their sum.

### 2.8 Free amino acid content

The free amino acid content in mustard leaf tea was determined by spectrophotometry according to the GB/T 8314–2013 standard, using glutamic acid as the reference solution. A 0.03 g sample of powdered mustard leaf was mixed with 5 mL of ddH_2_O and heated in a boiling water bath for 45 min. After extraction, 1 mL of the solution was combined with 0.5 mL of phosphate buffer (pH 8.0) and 0.5 mL of ninhydrin ethanolic solution, and the mixture was maintained at 100 °C for 15 min. The solution was then diluted to a final volume of 25 mL with ddH_2_O, and absorbance was measured at 570 nm using a UV-1800 spectrophotometer. The amount of free amino acid was calculated using glutamic acid (HPLC≥98 %, Yuanye Bio-Technology Co., Ltd., Shanghai, China) as a standard.

### 2.9 Ascorbic acid content

The ascorbic acid content was determined according to the previous report (Sun et al., 2018). Fifty milligrams of sample powder was homogenized and extracted with 5 mL 1.0 % oxalic acid, subsequently centrifuged 5 min at 4000 *g*. Each sample was filtered through a 0.22 μm cellulose acetate filter. HPLC analysis of ascorbic acid was carried out using an Agilent 1260 instrument with a VWD detector (Agilent Technologies, Inc., Palo Alto, USA). Samples (10 μL) were separated at 30 °C on a Waters Spherisorb C18 column (150 × 4.6 mm i.d.; 5 μm particle size), using a solvent of 0.1 % oxalic acid at a flow rate of 1.0 mL min^−1^. The amount of ascorbic acid was calculated from absorbance values at 243 nm, using ascorbic acid (HPLC≥99 %) (Sangon Biotech Co., Ltd., Shanghai, China) as a standard.

### 2.10 Total phenolics content

The total phenolics content was determined according to the previous report (Sun et al., 2018). Fifty milligrams of sample powder was extracted with 15 mL of 50 % ethanol and incubated at room temperature for 24 h in the dark. The suspension was centrifuged at 4000 *g* for 5 min at room temperature. Subsequently, 0.3 mL of the supernatant was combined with 1.5 mL of 0.2 M Folin-Ciocalteu reagent, and 1.2 mL of saturated sodium carbonate was added after 3 min. The mixtures were allowed to stand for 20 min at room temperature, and the absorbance was measured at 760 nm using a UV-1800 spectrophotometer. The amount of total phenolics was calculated using gallic acid (HPLC≥98 %, Yuanye Bio-Technology Co., Ltd., Shanghai, China) as a standard, and the results were expressed as mg garlic acid equivalent per g dry weight.

### 2.11 Ferric reducing antioxidant power (FRAP)

The sample powder (50 mg) was extracted with 3 mL of 50 % ethanol and incubated at room temperature for 24 h in the dark. Subsequently, 0.3 mL of the extract was combined with 2.7 mL of the FRAP working solution and incubated at 37 °C for 10 min. The absorbance was recorded at 593 nm using a spectrophotometer and FeSO₄·7H₂O (Beijing Coolaber Science & Technology Co., Ltd., Beijing China) as a standard. The results were expressed as μmol g^−1^ of dry weight (Sun et al., 2018).

### 2.12 2,2-azinobis (3-ethyl-benzothiazoline-6-sulfonic acid) (ABTS^+^) assay

ABTS^+^ radical cation scavenging capacity was performed according to the previous report (Sun et al., 2018). The sample powder (0.05 g) was extracted with 3 mL 50 % ethanol and incubated at room temperature for 24 h in the dark. Then 0.3 mL of extracts was added to 3 mL ABTS^+^ working solution and incubated for 2 h. The absorbance was then recorded at 734 nm using a spectrophotometer and Trolox (Beijing Solarbio Science & Technology Co., Beijing China) as a standard.

### 2.13 Statistical analysis

All assays were performed in triplicate. Results are presented as the mean ± standard deviation (SD). Statistical analyses were performed using IBM SPSS Statistics 21. For hypothesis testing, the null hypothesis (H₀) was defined as no significant differences between experimental groups. Differences were evaluated using the Least Significant Difference (LSD) test, with a statistical significance threshold set at *p* < 0.05 for rejecting H₀. Multivariate analyses, including Principal Component Analysis (PCA) and time-related trajectory analysis, were performed using SIMCA 13.0.

## 3 Results

### 3.1 Mustard tea raw material screening by sensory evaluation and nutritional content

**Fig. 1:**
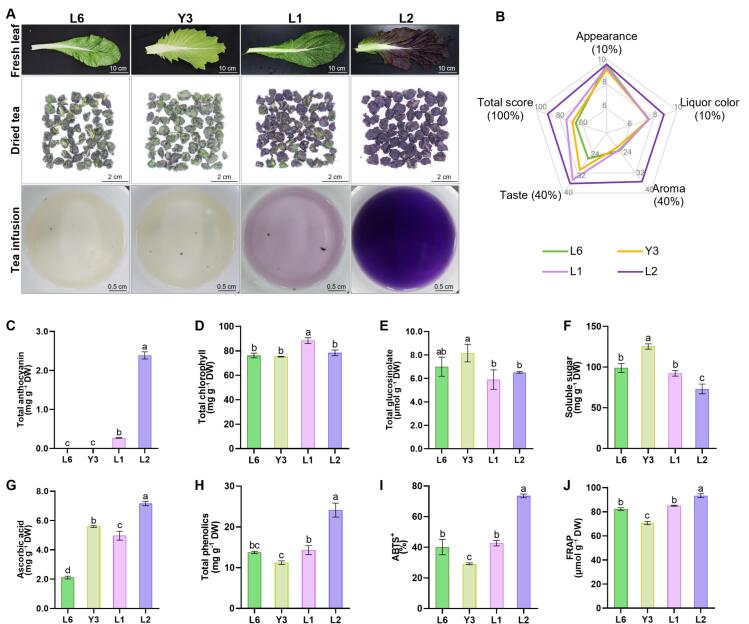
The sensory evaluation and nutrient compounds of the mustard leaf teas produced by four leaf mustard varieties. (A) Appearance of fresh leaf, dried tea and tea infusion of four varieties of leaf mustard; (B) Sensory evaluation of mustard teas produced by four varieties of leaf mustard; (C)-(J) The content of total anthocyanin (C), total chlorophyll (D), total glucosinolate (E), soluble sugar (F), ascorbic acid (G), total phenolics (H), and the level of FRAP (I) and ABTS^+^ in the four dried mustard tea. Different letters above the bars indicate significant differences (*P* < 0.05), the same as below. Differences were evaluated using the Least Significant Difference (LSD) test.

Four leaf mustard varieties with different leaf colors were utilized to produce mustard leaf tea using traditional green tea manufacturing techniques (Fig. 1A). Among the varieties, the sensory evaluation score for the tea made from purple leaf mustard (L2) was the highest (Fig. 1B). Its liquor was a bright purple, accompanied by a clean and sweet aroma, and a thick, mellow, and smooth taste. In contrast, the L6, Y3, and L1 teas presented a pronounced vegetable flavor with a bitter aroma or a strong pungent (Table S1).

Additionally, various nutritional components of dried mustard tea were analyzed. Notably, the anthocyanin content of the L2 dried tea reached 23.86 mg g^−1^, while undetected in L6 and Y3 (Fig. 1C). L1 tea exhibited the highest chlorophyll content, while Y3 tea had the highest GSL and soluble sugar contents (Fig. 1D-F). The ascorbic acid, total phenolics content, and antioxidant capacity of L2 dried mustard tea were higher than those in the other three varieties (Fig. 1G-J). Overall, mustard tea produced from L2 demonstrated a satisfactory sensory profile, high levels of bioactive components, and strong antioxidant capacity, indicating that purple leaf mustard L2 is a high-quality raw material for mustard tea preparation.

### 3.2 Sensory evaluation of mustard tea processed by different techniques

**Fig. 2:**
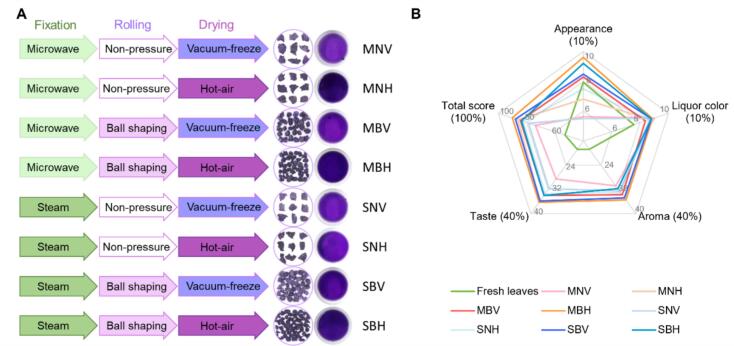
The manufacturing processes and sensory evaluation for purple mustard leaf tea. (A) Outlines of manufacturing processes for eight types of mustard leaf tea; (B) The sensory evaluation of mustard leaf tea produced by different manufacturing processes. (For interpretation of the references to color in this figure legend, the reader is referred to the web version of this article.)

To evaluate processing effects on purple-leaf mustard tea sensory, eight combinations of fixation (steaming/microwaving), rolling (pressure/non-pressure), and drying (vacuum-freezing/hot-air) methods were compared (Fig. 2A). These teas exhibited notable variations in appearance and flavor quality. The appearance score for the mustard tea produced by non-pressure rolling was low due to its thin, flaky form, which was prone to breakage during storage, significantly affecting the uniformity of the tea's morphology (Fig. 2A, S1). MNV showed the best liquor color (though not significantly different from others). The three process methods of mustard tea - MBH, MNH, and SBV - exhibited optimal aroma (clean, sweet with vegetable notes) creating a coordinated and pleasant experience with lasting fragrance. The MBH tea received the highest taste score, noted for its sweetness, mellowness, and refreshing quality, followed by SBV and MNH, with no significant differences (Fig. 2B, Table S2). The total sensory evaluation scores ranked MBH (90.23), SBV (87.83) and MNH (86.28) as statistically equivalent top performers, followed by MBV, SBH, SNH, SNV and MNV.

### 3.3 Health-promoting compounds and antioxidant activities analysis of mustard tea processed by different techniques

To further evaluate the effects of different processing techniques on the nutritional quality of mustard tea, the health-promoting compounds and antioxidant activities of four types of mustard tea—MBV, MBH, SBV, and SBH—were analyzed. The remaining four types of mustard tea produced by non-pressure rolling were excluded due to significant defects in the appearance of the dried tea.

#### 3.3.1 Anthocyanin content

**Fig. 3:**
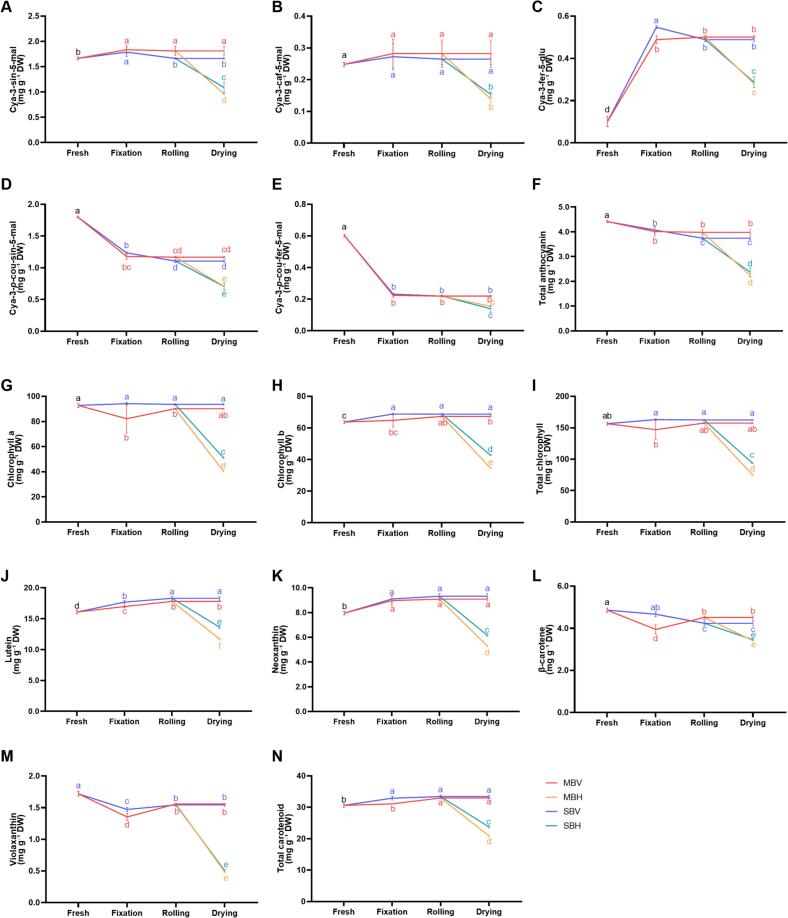
Anthocyanins, Chlorophyll and Carotenoids contents of mustard leaf tea processed by different methods. (A) Cya-3-sin-5-mal; (B) Cya-3-caf-5-mal; (C) Cya-3-fer-5-glu; (D) Cya-3-*p*-cou-sin-5-mal; (E) Cya-3-*p*-cou-fer-5-mal; (F) Total anthocyanins; (G) Chlorophyll a; (H) Chlorophyll b; (I) Total chlorophyll; (J) Lutein; (K) Neoxanthin; (L) β-carotene; (M) Violaxanthin; (N) Total carotenoids. Each data point represents the mean ± SD of triple biological replicates.

Anthocyanins are important nutrient compounds and the main coloring substance in purple mustard leaf tea. Five major anthocyanins were identified in the mustard tea according the relative retention time of the chromatography (Fig. 3A-E, S2A): Cyanidin 3-sinapoylferuloylsophoroside-5-malonylglucoside (Cya-3-sin-5-mal), Cyanidin 3-caffeoylferuloylsophoroside-5-malonylglucoside (Cya-3-caf-5-mal), Cyanidin 3-feruloylsophoroside-5-glucoside (Cya-3-fer-5-glu), Cyanidin 3-p-coumaroylsinapoylsophoroside-5-malonylglucoside (Cya-3-p-cou-sin-5-mal), and Cyanidin 3-p-coumaroylferuloylsophoroside-5-malonylglucoside (Cya-3-p-cou-fer-5-mal). Among these, Cya-3-sin-5-mal and Cya-3-p-cou-sin-5-mal were the most abundant, collectively accounting for approximately 80 % of the total anthocyanins. The fixation process enhanced the accumulation of Cya-3-sin-5-mal. Compared to the fresh sample, the levels of Cya-3-sin-5-mal increased by 7.44 % and 10.34 % with steaming and microwaving fixation, respectively. In contrast, hot-air drying significantly degraded Cya-3-sin-5-mal, reducing its content to about half of that obtained through vacuum-freezing drying (Fig. 3A). Similarly, the contents of Cya-3-caf-5-mal and Cya-3-fer-5-glu initially increased before declining during mustard tea processing (Fig. 3B-C). Additionally, the fixation process led to the degradation of Cya-3-p-cou-sin-5-mal and Cya-3-p-cou-fer-5-mal, with no significant difference observed between steaming and microwaving fixation (Fig. 3D-E). The total contents of all five anthocyanins decreased significantly with hot-air drying; compared to vacuum-freezing drying, hot-air drying reduced the total anthocyanin content of mustard tea by 36.65 % to 43.75 %. Ultimately, MBV exhibited the highest total anthocyanin content at 3.98 mg g^−1^, followed by SBV at 3.74 mg g^−1^ (Fig. 3F). The drying method had the most significant impact on anthocyanin content.

#### 3.3.2 Chlorophyll and carotenoid content

A total of two chlorophylls and four carotenoids were identified in purple mustard tea (Fig. 3G-N, S2B). There was no significant difference in the total chlorophyll content of SBV and MBV mustard teas compared to the fresh sample. In contrast, the chlorophyll contents of SBH and MBH decreased by 40.02 % and 51.97 %, respectively, compared to the fresh mustard sample (Fig. 3I). The fixation process enhanced the accumulation of carotenoids, specifically lutein and neoxanthin, while reducing the contents of β-carotene and violaxanthin (Fig. 3J-M). Steaming fixation proved more effective than microwaving, as it retained a higher concentration of carotenoids in the mustard tea (Fig. 3N). Consistent with the anthocyanins and chlorophyll, hot-air drying resulted in a sharp decrease in the levels of all four carotenoids, particularly violaxanthin, whose content fell to less than one-third of that through vacuum-freezing drying. Overall, SBV exhibited the highest total carotenoids content, followed by MBV, with SBH and MBH displaying lower levels (Fig. 3N).

#### 3.3.3 GSL composition and content

**Fig. 4:**
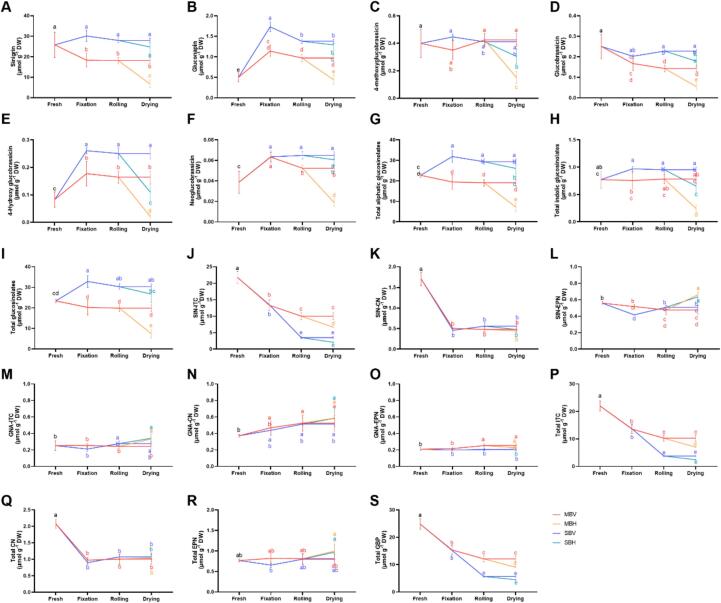
GSL and their breakdown products contents of mustard leaf tea processed by different methods. (A) sinigrin; (B) gluconapin; (C) 4-methoxyglucobrassicin; (D) glucobrassicin; (E) 4-hydroxy glucobrassicin; (F) neoglucobrassicin; (G) total aliphatic glucosinolate; (H) total indolic glucosinolate; (I) total glucosinolate; (J) SIN-ITC; (K) SIN-CN; (L) SIN-EPN; (M) GNA-ITC; (N) GNA-CN; (O) GNA-EPN; (P) Total ITC; (Q) Total CN; (R) Total EPN; (S) Total GBP.

Two aliphatic GSLs and four indolic GSLs were identified in mustard teas produced using four different processing methods (Fig. 4, S2C). Among these, sinigrin was the most abundant in fresh mustard (Fig. 4A). The sinigrin content decreased by 29.00 % after microwave fixation; however, steaming effectively prevented its degradation, resulting in no significant difference compared to the fresh sample. In contrast, rolling had no significant effect on sinigrin levels, while hot-air drying significantly promoted its degradation (Fig. 4A). Additionally, the content of gluconapin increased after fixation through steaming or microwaving, being 3.47-fold and 2.28-fold higher than that of the fresh leaves, respectively. However, gluconapin levels subsequently decreased during the rolling and drying processes. Among the four mustard teas, SBV exhibited the highest gluconapin content (Fig. 4B). Consistent with sinigrin, both 4-methoxyglucobrassicin and glucobrassicin contents decreased due to hot-air drying (Fig. 4C-D). The contents of 4-hydroxyglucobrassicin and neoglucobrassicin increased following fixation and decreased with hot-air drying, with the highest levels observed in SBV and the lowest in MBH (Fig. 4E-F). In summary, steaming fixation enhanced the levels of aliphatic and indolic GSLs, whereas microwaving fixation led to a decrease. Vacuum-freezing drying effectively preserved both the GSL composition and content (Fig. 4G-I).

#### 3.3.4 GBP composition and content

A total of six GBPs were identified in mustard, comprising two nitriles (CNs), two isothiocyanates (ITCs), and two epithionitriles (EPNs) (Fig. 4J-S). The content of 2-propenyl isothiocyanate (SIN-ITC) consistently decreased during the processing of mustard leaf tea, with the lowest levels observed in SBH, which was less than one-tenth of that in the fresh sample (Fig. 4J). Both steaming and microwaving fixation significantly enhanced the degradation of 3-butenenitrile (SIN-CN), whereas rolling and drying did not produce noticeable changes in its content (Fig. 4K). The 3,4-epithiobutanenitrile (SIN-EPN) content significantly increased with hot-air drying (Fig. 4L). The trends in 3-butenyl isothiocyanate (GNA-ITC) content during mustard tea processing mirrored those of SIN-EPN (Fig. 4M). Key steps in the mustard tea processing—fixation, rolling, and drying—promoted the accumulation of 4-pentenenitrile (GNA-CN), with significantly higher levels found in MBV, MBH, and SBH compared to the fresh sample (Fig. 4N). 4,5-epithiopentanenitrile (GNA-EPN) remained relatively stable throughout processing, exhibiting only slight changes in content (Fig. 4O). Overall, the processing of mustard tea resulted in the degradation of CNs, ITCs, and total GBP, with the total GBP content in the tea samples being only 18.04 % to 48.72 % of that in the fresh sample (Fig. 4P-S).

#### 3.3.5 Soluble sugars

**Fig. 5:**
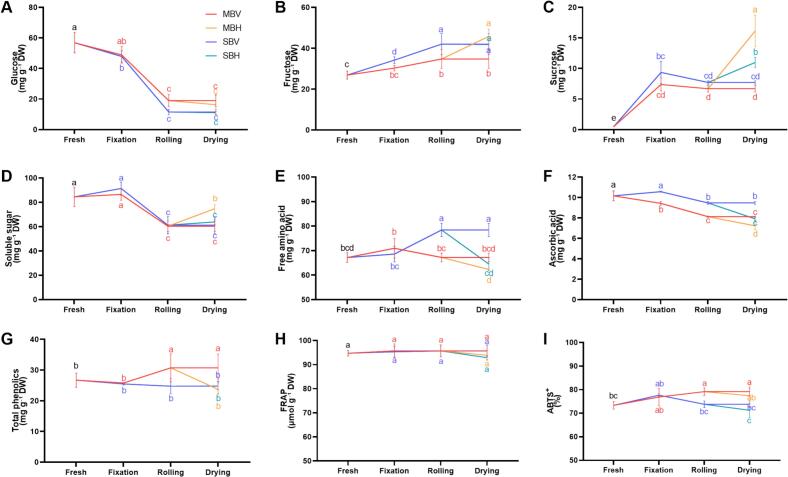
Soluble sugar (A-D), free amino acid (E), ascorbic acid (F), total phenolics (G) content and antioxidant capacity levels (H—I) of mustard leaf tea processed by different methods.

Three soluble sugars were identified in mustard: glucose, fructose, and sucrose (Fig. S2D). Among these, glucose was the most abundant, measuring 56.85 mg g^−1^ in fresh samples (Fig. 5A). Processing led to a continuous decrease in glucose content, particularly during the fixation and rolling stages, where reductions exceeded 60 %. However, no significant differences were observed in glucose levels across the four processing methods (Fig. 5A). The fructose content increased throughout the tea manufacturing processes, with values in MBH, SBV, SBH, and MBV being 1.71-, 1.57-, 1.57-, and 1.29- folds higher that of the fresh sample, respectively (Fig. 5B). Fixation and hot-air drying promoted the accumulation of sucrose, with the sucrose content in mustard tea dried by hot air being significantly higher than that dried by vacuum-freezing; specifically, MBH contained 2.42-fold higher sucrose than MBV (Fig. 5C). Among the mustard leaf teas, MBH exhibited the highest soluble sugar content, ranging from 1.45 to 1.92-fold higher than that of the other three types produced through different manufacturing processes (Fig. 5D).

#### 3.3.6 Free amino acid

Free amino acids are essential compounds contributing to the umami flavor of tea (Zhang et al., 2020). Consistent with most other nutrients, hot-air drying led to the degradation of amino acids, while vacuum-freezing drying effectively mitigated high-temperature damage to amino acids (Fig. 5E). The SBV method processed mustard leaf tea with the highest amino acid content, demonstrating statistically significant 1.17- to 1.26-fold increases compared to other processing methods. Consistent with sensory evaluation results, SBV is characterized by pronounced mellow and umami notes. This sensory profile likely correlates with its significantly elevated free amino acid content.

#### 3.3.7 Antioxidants and antioxidant capacity

Microwaving significantly induced the degradation of ascorbic acid in mustard leaves, resulting in a 7.22 % decrease in content compared to the fresh sample. In contrast, steaming fixation effectively prevented the degradation of ascorbic acid, maintaining levels similar to those of the fresh sample. Subsequently, during the rolling and drying processes, the ascorbic acid content continued to decline. The SBV treatment retained the most ascorbic acid, and the ascorbic acid content was significantly higher than MBH, MBV, and SBH (Fig. 5F, S2E).

The total phenolics content in fresh purple-leaf mustard was as high as 26.72 mg g^−1^ DW. All four processing methods effectively preserved the total phenolics content in mustard tea. The total phenolics content of MBH, SBV, and SBH did not significantly differ from that of the fresh sample, while MBV exhibited a significant increase of 1.15-fold compared to the fresh sample (Fig. 5G).

The antioxidant capacity was assessed using the FRAP and ABTS^+^ assays (Fig. 5H-I). Although hot-air drying decreased the FRAP levels of mustard leaf tea, no significant differences were observed among the teas produced by the four processing methods. Overall, the antioxidant capacity of mustard leaves did not change significantly during the tea manufacturing processes.

### 3.4 Time-related trajectory analysis

**Fig. 6:**
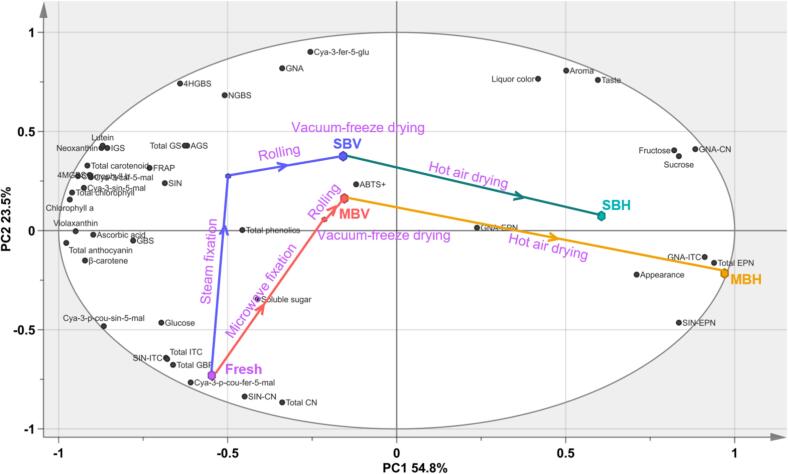
Time-related trajectory analysis of mustard leaf tea processed by different methods.

A time-related trajectory analysis separated different stages and methods of mustard leaf tea processing (Fig. 6). The first principal component (PC1) and the second principal component (PC2) accounted for 54.8 % and 23.5 % of the variance, respectively. PC1 exhibited a positive correlation with sensory evaluation indices, fructose, sucrose, and GNA-CN, while showing a negative correlation with total chlorophyll, carotenoids, anthocyanins, and GBPs. Throughout the mustard leaf tea processing, the disparity in nutritional component content between the mustard and the fresh sample progressively increased. Most phytochemical components were continuously lost during tea processing, particularly during fixation and hot-air drying, which were critical steps that contributed to the differences in metabolites of mustard leaf tea. Among the four manufacturing processes, MBV and SBV were closest in distance to the fresh mustard leaves, sharing similar compositions and phytochemical content with the fresh sample. In contrast, MBH exhibited the greatest distance from the fresh sample, indicating the most significant difference in phytochemical components.

### 3.5 Mustard leaf tea blended with traditional Chinese teas

**Fig. 7:**
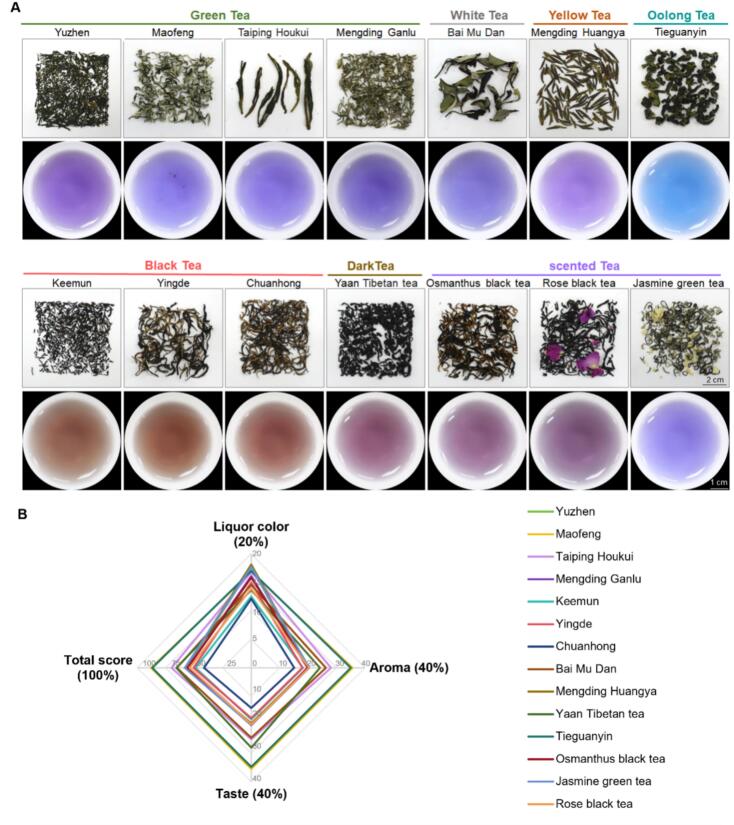
The purple mustard leaf tea blended with fourteen traditional Chinese teas. (A) The appearance of fourteen traditional Chinese teas and the tea infusion of the fourteen traditional Chinese teas brewed after being blended with mustard leaf tea in a 1:1; (B) The sensory evaluation of the mustard leaf teas blended with fourteen traditional Chinese teas. (For interpretation of the references to color in this figure legend, the reader is referred to the web version of this article.)

Although SBV effectively preserves the nutritional quality of mustard, its aroma and taste are slightly inferior to MBH. To retain more original metabolites from mustard leaves while enhancing the sensory experience for tea enthusiasts, mustard leaf tea was blended with traditional Chinese teas to identify combinations with a pleasing aroma and coordinated flavor. A total of 14 typical and mainstream teas were screened for brewing in a 1:1 ratio with mustard tea (Fig. 7A). Ten scholars specializing in tea science conducted a comprehensive evaluation. Mustard leaf tea blended with Maofeng green tea and Tieguanyin oolong tea received the highest scores of 87.93 and 87.07, significantly surpassing those of the other 12 teas (Fig. 7B). When blended with Maofeng green tea, the liquor exhibited a bright purple-blue hue, and the combination of mustard and tea created a pleasant aroma. The flavors were fresh, sweet, thick, mellow, and taste coordinated. When blended with Tieguanyin oolong tea, the liquor appeared bright light blue and had a fragrant, lasting aroma characterized by clean and refreshing notes of orchids and vegetables. The flavor was mellow and sweet, with slightly grassy and astringent undertones (Table S3). Overall, blending with other tea leaves significantly enriched the taste of mustard tea.

## 4 Discussion

The plant species commonly used for herbal teas may be derived from medicinal plants or fragrant flowers and fruits (Bai et al., 2023; Liu et al., 2023). However, using medicinal plants to make herbal teas may pose safety risks (Halegoua-DeMarzio & Navarro, 2024; Wiedenfeld, 2011). Additionally, many medicinal plants have a pronounced bitter taste, affecting palatability. Therefore, new herbal tea products should be developed using non-toxic species for long-term consumption, rich in health-promoting compounds and possessing acceptable flavors, such as vegetables instead of medicinal plants, to meet consumer demands for both sensory and nutritional quality in herbal tea. Leaf mustard contains numerous nutrients and bioactive substances, including ascorbic acid, carotenoids, various phenolics, and GSLs (Di et al., 2023; Zhang et al., 2021). Notably, GSLs, which are uniquely distributed within the order Brassicales, contribute to mustard's distinctive fragrance. Furthermore, the hydrolysis products of GSLs have been recognized for their antioxidant properties and potential to lower glucose levels and reduce cancer incidence (Jo et al., 2018; Sundaram et al., 2022). Leaf mustard meets safety and health criteria for herbal tea raw materials. However, the potential of mustard leaves as a raw material for herbal tea remains unexplored. In this study, the development of a novel herbal tea from purple leaf mustard was reported. The raw material and processing technology of mustard tea were screened to retain the nutrient content of mustard and sensory quality. Then, it was blended with traditional teas to further enhance its flavor.

Variety is a crucial factor influencing the flavor and quality of tea. The production of many famous teas has certain requirements for varieties (Feng et al., 2024). In this study, four different color varieties of leaf mustard were processed using traditional green tea technology. The mustard leaf tea produced from the L2 variety excelled in both sensory evaluation and nutritional quality. Its high anthocyanin content contributed to the deep purple and shiny color of the infusion (Fig. 1A), which was markedly distinct from the well-known purple foliage teas of *Camellia sinensis*, namely ‘Ziyan’ and ‘Zijuan’ (Li et al., 2021). This distinction is likely due to the total anthocyanin content of L2 (4.42 mg g^−1^ DW), which is higher than that of ‘Ziyan’ (1.61 mg g^−1^ DW) and ‘Zijuan’ (2.86 mg g^−1^ DW). The main anthocyanin component in mustard L2 was cyanidin (Fig. 3A-F), whereas ‘Ziyan’ and ‘Zijuan’ predominantly contained delphinidin (Tan et al., 2023). The anthocyanin content in mustard tea was also significantly greater than that found in most of herbal teas, such as mulberry and rose (Liu et al., 2023; Yang, Yang, & Zheng, 2010). Furthermore, tea from the L2 exhibited the highest antioxidant levels, suggesting that mustard is a viable candidate for herbal tea production and that purple leaf mustard L2 is an excellent raw material for tea processing.

The flavor and quality of tea products vary based on the raw materials used and the manufacturing processes employed (Zhu et al., 2021). In this study, mustard leaf tea was processed using green tea technology to preserve more original metabolites, focusing on the key steps of the process, which involved fixation, rolling/shaping, and drying. The fixation stage involves heating the fresh leaves to rapidly inactivate endogenous enzymes (Zhang et al., 2019). Common methods for fixing green tea include steaming, pan-frying, and microwaving (Wang et al., 2020). Previous studies have shown that steaming fixation better maintains the “three green” characteristics—green dry tea leaves, green tea infusion, and green infused leaves and the aroma concentrations of steamed teas were significantly higher than those of pan-fried teas (Wang et al., 2022; Xue et al., 2023). While, microwaving fixation tends to enhance the levels of free amino acids, resulting in a sweet and mellow taste (Zhu et al., 2022). Consistent with previous studies, fresh L2 leaves fixed by steaming exhibited higher chlorophyll and carotenoid content (Fig. 3G-N) but slightly lower amino acid levels compared to those fixed by microwaving (Fig. 5E), suggesting similar metabolic changes occur during the fixation of mustard leaves as with *Camellia sinensis* (Xue et al., 2023; Zhu et al., 2022). Additionally, steaming fixation prevented the degradation of ascorbic acid (Fig. 5F) and promoted the accumulation of GSLs, which was consistent with previous studies. Steaming is the most effective method for retaining GSL content in cruciferous vegetables compared to blanching, boiling, or microwaving, and GSL was metabolized into compounds that exhibit anti-carcinogenic and anti-inflammatory properties by the gut microbiota (Mullaney, Kelly, McGhie, Ansell, & Heyes, 2013; Sikorska-Zimny & Beneduce, 2021). These results indicate that steaming fixation is superior to microwaving fixation in preserving the original metabolites of mustard.

The leaf-rolling process disrupts the intracellular structure, facilitating the outflow of cell sap (Miyagishima et al., 2011). Compared to drying and fixation, rolling has the least impact on the non-volatile and aroma components of tea (Sun et al., 2023; Wang et al., 2021). Consistent with previous studies, the content of most health-promoting compounds remained relatively unchanged after rolling during the processing of mustard leaf tea (Fig. 6). Another advantage of rolling is that it shapes tea leaves, making them more compact for storage (Zhou et al., 2024). In this study, tea leaves processed using non-pressure rolling were irregularly flaky and fragile during storage and transportation, which hindered factory production. Conversely, mustard leaf tea rolled into small balls exhibited a neat and compact appearance, enhancing its marketability (Fig. 2A). Nevertheless, the rolling method and duration of mustard tea require further investigation.

Drying is a crucial step in tea processing, promoting the formation of aroma, taste, and color (Wang et al., 2022). However, high temperatures during drying can lead to excessive nutrient loss in tea (Han et al., 2023; Qu et al., 2019). In recent years, vacuum freeze-drying patterns gradually emerged (Liu et al., 2023). The lower temperatures involved in this process allow for maximal retention of nutrients and bioactive compounds (Bhatta, Stevanovic Janezic, & Ratti, 2020). For example, rose tea processed via vacuum freeze-drying exhibited higher contents of anthocyanins, proanthocyanidins, and total phenolics compared to those obtained through normal-temperature drying and hot-air drying (Liu et al., 2023). Higher drying temperatures typically result in increased bitterness and astringency in green tea (Wang et al., 2022). In this study, hot-air drying resulted in a significant loss of nutrients in mustard leaf tea. Consistent with the previous research, the contents of anthocyanins, chlorophyll, carotenoids, GSLs, ascorbic acid, and amino acids in mustard leaf tea processed by hot-air drying were significantly lower than those in tea processed via vacuum freeze-drying (Han et al., 2023; Qu et al., 2019). Robbins et al. (2011) reported that freeze-dried Brussels sprouts contained higher concentrations of GSLs and isothiocyanates compared to oven-dried Brussels sprouts. Surprisingly, sucrose was significantly accumulated in mustard leaf tea processed by hot-air drying, possibly due to high temperatures promoting the hydrolysis of polysaccharides (Fig. 5C). The increased soluble sugar content may enhance the flavor of the mustard leaf tea of MBH.

**Fig. 8:**
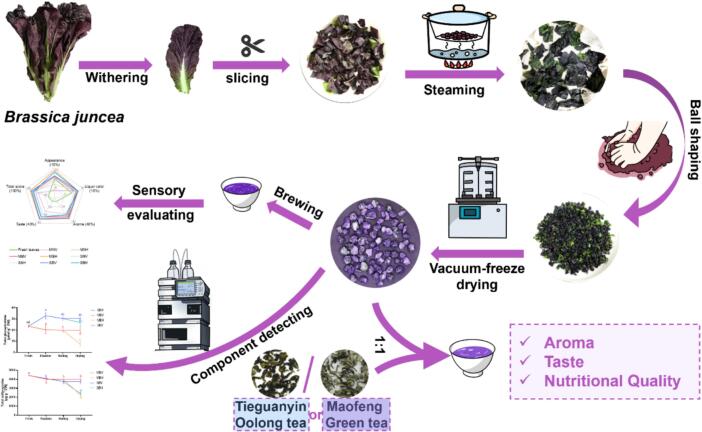
The model illustrating the manufacturing process of purple mustard leaf tea and the blended teas produced by mustard leaf tea mixed with Tieguanyin oolong tea or Maofeng green tea. (For interpretation of the references to color in this figure legend, the reader is referred to the web version of this article.)

In the tea manufacturing process, the interactions between processing steps greatly influence the flavor and quality of the final product. The top two total scores in the sensory evaluation of mustard leaf tea were awarded to the mustard tea of MBH and SBV process by experts in tea science. There was a high degree of synergy between microwaving fixation and hot-air drying, resulting in mustard leaf tea with a clean and sweet aroma, which may be caused by MBH having a high soluble sugar content and a low GSL content. In contrast, steaming fixation combined with vacuum freeze-drying produced mustard leaf tea with a clean and refreshing aroma, albeit with less sweetness than MBH. However, the sensory advantages of MBH do not sufficiently compensate for the nutritional losses, particularly concerning the health-promoting compounds such as anthocyanins, carotenoids, and GSLs. Previous studies have reported that GSLs contribute to a pungent and bitter taste, as well as a sulfurous aroma, particularly isothiocyanates derived from sinigrin and progoitrin, which are responsible for bitterness (Prieto, López, & Simal-Gandara, 2019), while those from gluconapin and glucobrassicanapin contribute to a pungent flavor (Juge, Mithen, & Traka, 2007; Rosa, Heaney, Fenwick, Portas, & Janick, 1996). This may explain why SBV exhibited a certain bitterness and spiciness in its flavor. Considering all factors, the optimal production technology for mustard leaf tea involves steaming fixation followed by rolling and then vacuum freeze-drying (Fig. 8). Furthermore, optimizing the processing conditions could further enhance the flavor and quality of mustard leaf tea.

To enhance the flavor of tea, it is frequently blended with flowers, herbs, or other substances to create a coordinated and richly layered taste profile, as exemplified by jasmine tea and Earl Grey tea (An et al., 2022; Kim, Cha, Shin, & Chun, 2018). In this study, two teas exhibited remarkably coordinated aromas and flavors when combined with mustard leaf tea: Maofeng green tea and Tieguanyin oolong tea (Fig. 7). Interestingly, the blended teas displayed a variety of vibrant colors, ranging from purple and indigo to reddish-brown, potentially due to the varying colors of anthocyanins at different pH levels (Sui, Bary, & Zhou, 2016). Blending mustard leaf tea with preferred teas or herbs can significantly enhance the aesthetic appeal for consumers, contributing to their overall enjoyment. Overall, purple mustard leaf tea demonstrates excellent sensory quality, a high concentration of health-promoting compounds, and an appealing color for blended teas, indicating its potential in the consumer market.

## 5 Conclusion

Herbal teas have gained popularity in many countries due to their appealing fragrances and health benefits. Recently, there has been increased interest in the functional, sensory, and nutritional qualities of herbal teas, indicating broad market prospects for their development. In this study, a novel purple mustard leaf tea using the green tea manufacturing process was developed. The mustard leaf tea produced through steaming fixation, rolling, and vacuum freeze-drying maximized nutrient and bioactive compound retention while achieving high sensory evaluations. The blended tea, which combines mustard leaf tea with Maofeng green tea or Tieguanyin oolong tea, exhibited coordinated aromas and flavors. However, the health benefits of long-term consumption of mustard leaf tea also remain to be verified. Additionally, this study has only preliminarily explored the blending effects of mustard leaf tea with traditional teas, and the optimal blending ratios and brewing methods still require further investigation.

## Ethics & standards requirements for sensory evaluation

Since the research study was not of a medical nature, and the raw material (mustard) was a vegetable that people consume daily, ethical permission was not required, in line with national regulations. Written informed consent was obtained from all participants. Participants could withdraw from the study at any time without giving a reason. The tested products were safe for consumption.

## CRediT authorship contribution statement

**Xuena Yu:** Writing – original draft, Investigation. **Hongmei Di:** Writing – original draft, Investigation. **Yatian Zhao:** Writing – original draft, Data curation. **Ruobin Liu:** Investigation. **Yiqing Wang:** Data curation. **Yuxin Lu:** Data curation. **Kehao Liang:** Data curation, Conceptualization. **Zhifeng Chen:** Data curation. **Jie Ma:** Funding acquisition, Data curation. **Zhongrong Guan:** Funding acquisition, Data curation. **Zhi Huang:** Investigation, Funding acquisition. **Yi Tang:** Data curation. **Jingyi Xu:** Investigation. **Huanxiu Li:** Investigation. **Qian Tang:** Investigation. **Yiting Chen:** Writing – review & editing. **Jinlin Bian:** Writing – original draft. **Fen Zhang:** Writing – review & editing. **Bo Sun:** Writing – review & editing, Funding acquisition.

## Declaration of competing interest

The authors declare that they have no known competing financial interests or personal relationships that could have appeared to influence the work reported in this paper.

## Data Availability

All data that support the results are included in this paper and within its supplementary data published online.

## Appendix A Supplementary data

Supplementary material Image 1
