# Supplementary material for: Optimized processing and formulation for retaining the sensory and nutritional quality of mustard leaf tea
Source: Food Chem X. 2025 May 1;27:102515. doi: 10.1016/j.fochx.2025.102515 (PMC12124665; doi:10.1016/j.fochx.2025.102515)
Supplement: Supplementary file 1 — Supplementary material [file mmc1.docx]

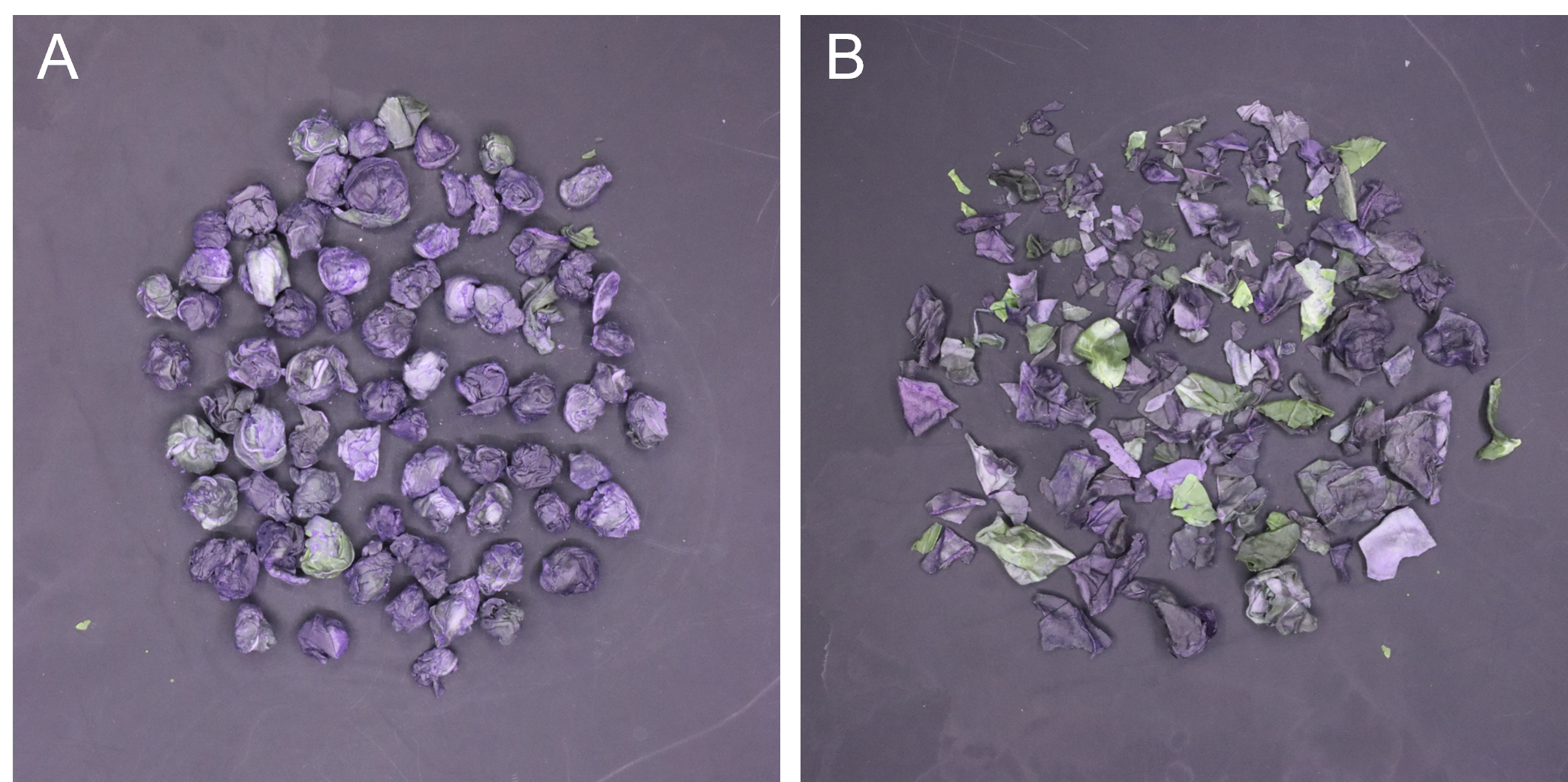


**Figure S1 The Typical appearance of ball-shaped mustard leaves tea (A) and non-pressure rolled tea (B) after storage for 3 months.**


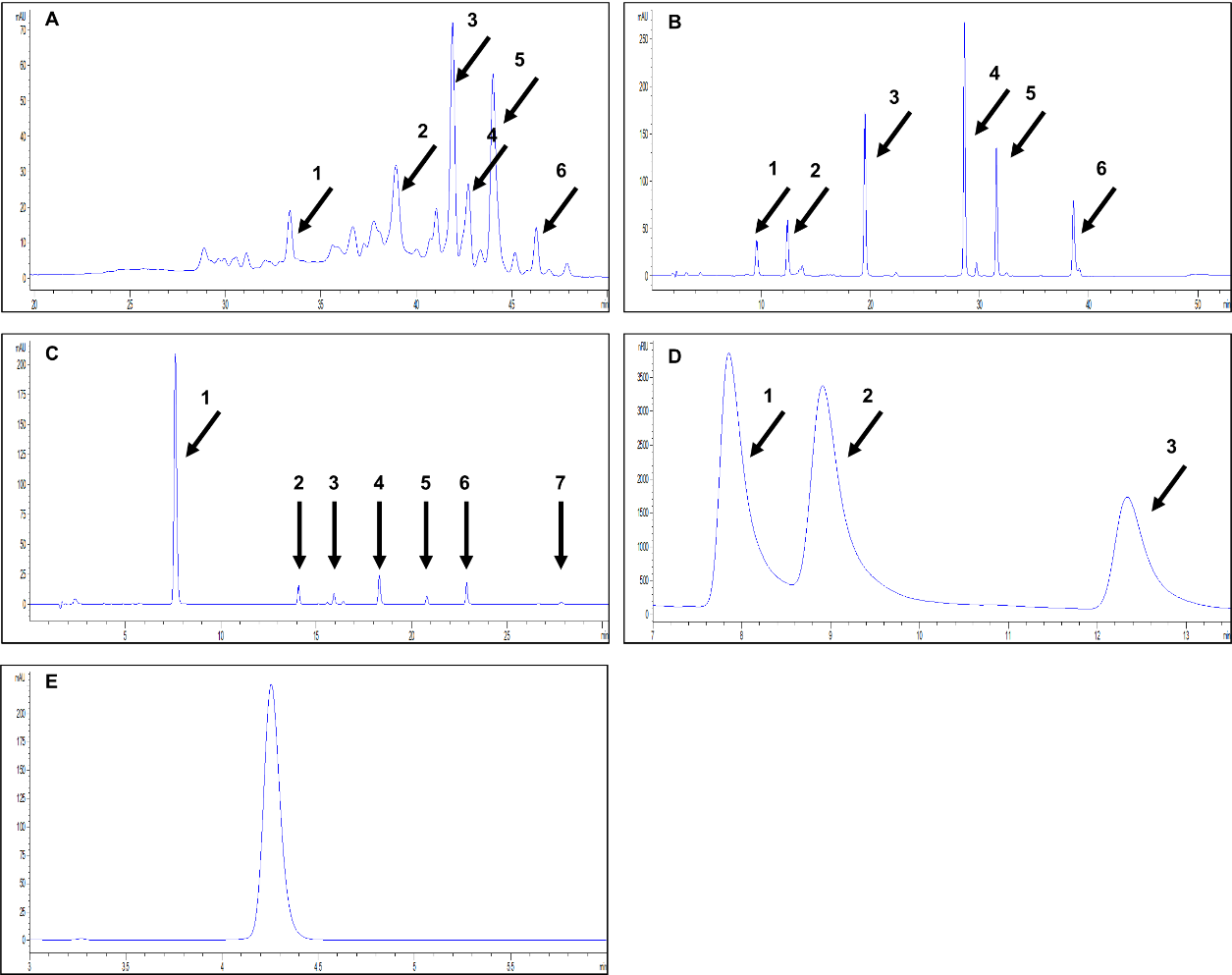


**Figure S2 HPLC of health-promoting compounds in the purple mustard leaf tea.**

(A) Anthocyanins, 1: Cyanidin 3-caffeoylferuloylsophoroside-5-malonyglucoside, 2, 3: Cyanidin 3-sinapoylferuloylsophoroside-5-malonylglucoside, 4: Cyanidin 3-feruloylsophoroside-5-glucoside, 5: Cyanidin 3-*p*-coumaroylsinapoylsophoroside-5- malonylglucoside, 6: Cyanidin 3-*p*-coumaroylferuloylsophoroside-5-malonylglucoside; (B) Chlorophyll and carotenoids, 1: Neoxanthin, 2: Violaxanthin, 3: Lutein, 4: Chlorophyll b, 5: Chlorophyll a, 6: β-carotene; (C) Glucosinolate, 1: Sinigrin, 2: Gluconapin, 3: 4-Hydroxy glucobrassicin, 4: ortho Nitrophenyl β-D-galactopyranoside, 5: Glucobrassicin, 6: 4-Methoxyglucobrassicin, 7: Neoglucobrassicin; (D) Soluble sugars, 1: Fructose, 2: Glucose, 3: Sucrose; (E) Ascorbic acid.

**Table S1 Sensory evaluation of different varieties of mustard leaves teas**

| **Sample** | **Appearance** | **Appearance score (10)** | **liquor color** | **liquor color score (10)** | **Aroma** | **Aroma score (40)** | **Taste** | **Taste score (40)** | **Total score (100)** |
| --- | --- | --- | --- | --- | --- | --- | --- | --- | --- |
| L6 | round and tight, delicate green, slightly mixed | 9.55±0.06 a | light yellow, bright and clear | 7.55±0.49 b | clean and refreshing, high vegetable smell with the of bitter melon, lasting | 23.00±1.41 b | mellow, thick and sweet after taste, distinctly vegetable flavor | 26.25±0.96 c | 66.35±2.57 c |
| Y3 | frog skin-like green and slightly bloom, slightly heavy | 9.15±0.13 c | light yellow, bright | 7.60±0.64 b | clean and refreshing with sweet, high and intensive aromas, offensive and disharmonious aromas | 21.75±1.26 b | mellow and thick, slightly sweet and brisk with vegetable flavor | 30.75±1.89 b | 69.25±2.35 c |
| L1 | fine round, heavy and neat, mixed with deep purple and green, litter bright | 9.35±0.13 b | light purple, clear and bright | 7.63±1.15 b | clean and refreshing with roasted, grassy and astringent, approach harmonious aromas | 22.75±1.71 b | sweet and mellow, brisk and smooth with grassy and astringent | 34.75±1.26 a | 74.48±3.93 b |
| L2 | fine round and slightly flat, even colour, heavy and even | 9.60±0.09 a | purple and shiny | 8.88±0.25 a | clean and refreshing, sweet aroma with litter vegetable smell, high sweetness, harmonious and pleasure | 35.50±1.91 a | sweet and mellow, thick and brisk, smooth aftertaste | 36.25±0.96 a | 90.23±2.92 a |

**Table S2 Sensory evaluation table of eight types of purple mustard leaves tea by different manufacturing process**

| **Sample** | **Appearance** | **Appearance score (10)** | **liquor color** | **liquor color score (10)** | **Aroma** | **Aroma score (40)** | **Taste** | **Taste score (40)** | **Total score (100)** |
| --- | --- | --- | --- | --- | --- | --- | --- | --- | --- |
| MNV | flakes and light, approach neat, purple with green | 5.63±0.48 e | light purple and bright | 9.00±0.82 a | clean and fresh, show vegetable smell, approach harmonious | 30.78±0.96 c | mellow and thick with sweet, show vegetable taste, better coordination | 28.50±1.91 d | 73.88±2.66 d |
| SNV | flakes and light, less neat, purple and green | 5.50±0.58 e | purple and bright | 8.85±1.03 a | clean and refreshing with sweet, approximate harmonious, show vegetable smell | 32.50±1.73 abc | mellow and thick with sweet, clean and brisk, show vegetable taste | 31.75±2.5 c | 78.60±4.95 c |
| MBV | round and uniform, approximately tight, dark purple with green | 8.28±0.63 b | purple and shiny | 8.38±1.25 a | clean with vegetable aroma, sweet and lasting, high aroma, approach coordinated and pleasure | 33.75±1.50 abc | mellow with vegetable taste, sweet and lasting aftertaste | 34.00±1.83 abc | 84.40±4.07 b |
| SBV | round and approximate heavy, litter flat and neat | 8.48±0.46 b | purple and bright | 8.80±0.24 a | clean and sweet with a vegetable smell, pleasant scent | 34.75±1.26 ab | mellow and thick, sweet and brisk, with vegetable flavor | 35.80±1.33 ab | 87.83±2.57 ab |
| MBH | fine round and litter flat, heavy and even, deep purple | 9.60±0.09 a | purple and shiny | 8.88±0.25 a | clean and high sweet with vegetable smell, pleasant and harmonious | 35.50±1.91 a | sweet, mellow, thick, brisk and delicate taste with vegetable flavor | 36.25±0.96 a | 90.23±2.92 a |
| MNH | flakes, approach neat with lumps of leaf, purple with green mixed | 6.80±0.27 d | purple and bright | 8.73±0.22 a | clean and sweet, superior vegetable aroma, high sweet with vegetable smell, high aroma | 35.00±2.16 a | sweet, mellow and thick with vegetable flavor | 35.75±0.96 ab | 86.28±2.60 ab |
| SBH | wiry, round and tight, heavy and even, deep purple | 9.20±0.14 a | purple and bright | 8.80±0.68 a | clean and sweet, the aroma of vegetable was weak in the early stage and prominent in the late stage, more lasting, approach coordinated | 31.75±2.87 bc | mellow and thick with sweet, and litter vegetable flavor | 34.00±0.82 abc | 83.75±2.15 b |
| SNH | flakes and neat, less even, purple with green mixed | 7.50±0.18 c | purple and bright | 8.63±0.86 a | clean and fresh with sweet, litter vegetable smell and weak aroma | 33.00±2.16 abc | mellow, thick, clean and brisk with litter vegetable flavor | 33.50±0.58 bc | 82.63±2.95 bc |

**Table S3 Sensory evaluation table of the purple mustard leaves tea blended with fourteen Chinese traditional teas**

| **Tea type** | **Tea variety** | **liquor color** | **liquor color score (20)** | **Aroma** | **Aroma score (40)** | **Taste** | **Taste score (40)** | **Total score (40)** |
| --- | --- | --- | --- | --- | --- | --- | --- | --- |
| Green tea | Yuzhen | Light purple, shiny | 17.60±0.53ab | clean and fresh, slightly vegetable flavor | 19.83±0.67def | Strong, mellow, thick, sweet and brisk, without show vegetable flavor | 19.77±1.27de | 57.20±2.40d |
| Green tea | Maofeng | violet, bright | 16.83±0.76bc | tend aroma, higher aroma, tea aroma work well with vegetable aroma, Pleasant | 35.60±1.25a | mellow, thick, fresh, sweet taste harmony with vegetable flavor | 35.50±0.60a | 87.93±1.16a |
| Green tea | Taiping Houkui | purple, bright | 16.83±0.91bc | flowery and clean aroma, high and lasting, more show vegetable flavor | 28.03±1.15b | clean and mellow, fresh and brisk, fresh and sweet taste with vegetable flavor | 25.13±1.11c | 70.00±2.98b |
| Green tea | Mengding Ganlu | purple, bright | 16.07±0.35cd | fresh and tend aroma with sweet chestnut aroma, high and lasting with slightly vegetable flavor | 19.80±0.62def | fresh, mellow, thick and brisk taste without vegetable flavor | 20.07±0.70d | 55.93±1.55de |
| Black tea | Keemun | light brown, approach bright | 12.50±0.79h | sweet and honey aroma, high, sharp and lasting less vegetable flavor | 18.20±1.11ef | sweet, mellow and thick, delicate taste without vegetable flavor | 18.10±0.95ef | 48.80±0.79f |
| Black tea | Yingde | tan, less bright | 14.93±0.21ef | high fresh and sweet, strong aroma, high and lasting, less vegetable flavor | 18.00±0.40f | sweet, strong mellow and thick taste without vegetable flavor | 17.70±0.44f | 50.63±0.40f |
| Black tea | Chuanhong | tan, less bright | 12.10±0.36h | sweet aroma and less vegetable flavor | 15.17±0.38g | mellow and thick with fresh and sweet taste without vegetable flavor | 14.13±0.96g | 41.40±0.53g |
| White tea | Bai Mu Dan | purple, brighter | 14.53±0.72fg | clean and fresh aroma with sweet, higher and more lasting with vegetable flavor | 26.27±1.32c | clean, fresh, sweet and brisk taste with vegetable flavor | 24.53±1.01c | 65.33±1.63c |
| Yellow tea | Mengding Huangya | purple, shiny | 18.10±0.56a | fresh and tender aroma, and not show vegetable flavor | 20.13±1.40e | strong, mellow and thick, sweet and brisk taste with slightly vegetable flavor | 19.50±0.56def | 57.73±2.31d |
| Dark tea | Yaan Tibetan tea | deep taro purplemore, bright | 13.67±0.49g | pure and normal, aroma after aging, show vegetable flavor | 24.17±0.86d | sweet and mellow taste with vegetable flavor | 28.00±0.90b | 65.83±1.36c |
| Oolong tea | Tieguanyin | wathet blue, bright | 17.00±0.20bc | clean and refreshing, orchid, fragrant and lasting, high aroma and show vegetable flavor | 34.97±1.75a | mellow, clean and sweet teast with litter astringent and vegetable flavor | 34.77±1.27a | 87.07±1.20a |
| Scented tea | Osmanthus black tea | deep taro purplemore, bright | 15.83±0.38de | sweet and show sweet-scented osmanthus without vegetable flavor | 19.90±0.75ef | mellow, thick, sweet and brisk taste with osmanthus flavor, no vegetable flavor | 20.17±1.86d | 55.90±1.45de |
| Scented tea | Jasmine green tea | purple, shiny | 17.70±0.26ab | Jasmine flowers scent, fresher lovely, high and heavy aroma, no vegetable flavor | 20.43±0.59e | strong, mellow and thick taste, sweet after taste, jasmine flavor with litter astringent, no vegetable flavor | 20.13±1.40d | 58.27±1.91d |
| Scented tea | Rose black tea | puce, brighter | 13.90±0.46g | sweet, fresher with rose scent, no vegetable flavor | 20.30±0.89e | mellow and sweet taste with rose flavor, more delicate taste, no vegetable flavor | 19.87±0.61de | 54.07±1.00e |
